# Interprofessional practice in health care: an educational project with four learning sequences for students from six study programs

**DOI:** 10.3205/zma001028

**Published:** 2016-04-29

**Authors:** Anna Christina Nowak, Kathrin Klimke-Jung, Thorsten Schäfer, Karl Reif

**Affiliations:** 1 Hochschule für Gesundheit, Department für Angewandte Gesundheitswissenschaften, Bochum, Germany; 2 Ruhr-Universität Bochum, Zentrum für Medizinische Lehre, Bochum, Germany

**Keywords:** Interprofessional education, interdisciplinary communication, learning context, project report

## Abstract

**Introduction:** In response to demographic changes and the growing complexity of healthcare demands, national and international organizations are requiring greater cooperation among the health professions. Implementation of interprofessional learning programs within study programs in medicine, midwifery, nursing, and therapy is still rare. The first projects are currently underway in Germany. This paper presents the experience gathered by the organizers as interprofessional courses for six study programs were implemented.

**Project description:** As part of the collaborative project “Interprofessional Practice in Health Care” between the Medical School at the Ruhr University in Bochum and the Department for Applied Health Sciences at the Hochschule für Gesundheit, interprofessional curricular units were developed, taught and evaluated with the aim of establishing permanent and joint curricular structures at the two German universities. Imparting communication skills, knowledge of and appreciation for the work performed by the other health professions, as well as having students reflect on their own professional roles and responsibilities, were the focus of four curricular units. Students worked together in small interprofessional groups.

**Results: **A total of 220 students enrolled in occupational therapy, midwifery, speech therapy, medicine, nursing, and physiotherapy participated in small-group seminars. When conducting and implementing the seminars, administrative and methodological challenges became apparent, and this should be taken into consideration in regard to any future development of interprofessional courses. Integration into existing curricula, along with finding time in the various schedules and appropriate classroom space for small groups, were among the challenges faced. For over 86% of the students it was important that students from all six of the degree programs involved participated in the project. A detailed analysis of the content and evaluation will follow.

**Conclusion: **The value of the project’s aim to include as many study programs in the health professions and medicine as possible was confirmed by the participating students. However, accomplishing this requires a substantial amount of organizational effort in terms of scheduling, finding classroom space and integration into existing curricula. Careful attention must be given specifically to the coordination of monoprofessional and interprofessional teaching units.

## Zusammenfassung

**Einleitung: **Vor dem Hintergrund des demographischen Wandels und der Zunahme komplexer Versorgungsanforderungen fordern nationale und internationale Organisationen eine verstärkte Kooperation der Gesundheitsberufe. Die Implementierung von interprofessionellen Lehrangeboten in die Studiengänge der medizinischen, geburtshilflichen, pflegerischen und therapeutischen Berufe ist noch selten. Derzeit werden in Deutschland erste Projekte erprobt. Im vorliegenden Artikel werden die Prozesserfahrungen der Organisatoren bei der Implementierung gemeinsamer interprofessioneller Lehrangebote für sechs Studiengänge dargestellt.

**Projektbeschreibung: **Im Rahmen des Kooperationsprojekts „Interprofessionelles Handeln im Gesundheitswesen“ zwischen der Medizinischen Fakultät der Ruhr-Universität Bochum und dem Department für Angewandte Gesundheitswissenschaften der Hochschule für Gesundheit wurden interprofessionelle Lerneinheiten entwickelt, implementiert und evaluiert mit dem Ziel, langfristige curriculare Ausbildungsstrukturen zwischen den beiden Hochschulen zu etablieren. In vier Lernsequenzen standen die Vermittlung von kommunikativen Kompetenzen, das Wissen und die Wertschätzung gegenüber den Tätigkeitsbereichen der anderen Gesundheitsberufe sowie die Reflexion der eigenen beruflichen Rolle und Verantwortungsbereiche im Vordergrund. Dazu arbeiteten die Studierenden in interprofessionellen Kleingruppen zusammen.

**Ergebnisse: **Insgesamt haben 220 Studierende der Ergotherapie, der Hebammenkunde, der Logopädie, der Medizin, der Pflege und der Physiotherapie an interprofessionellen Kleingruppenseminaren teilgenommen. Bei der Durchführung und Implementierung der Lehrveranstaltungen zeigten sich insbesondere strukturelle und methodische Herausforderungen, die bei der zukünftigen Entwicklung interprofessioneller Lehrangebote berücksichtigt werden sollten. Hierzu zählen die curriculare Einbettung, gemeinsame Zeiträume in den Stundenplänen und räumliche Ressourcen für Kleingruppenarbeit. Für über 86 Prozent der Studierenden war es wichtig, dass alle Studierenden der sechs Studiengänge am Projekt teilnehmen. Eine detaillierte inhaltliche Analyse und Evaluation wird folgen.

**Schlussfolgerung: **Das Projektziel der Einbeziehung möglichst vieler Studiengänge der Gesundheitsberufe und der Medizin wird von den teilnehmenden Studierenden bekräftigt. Dies erfordert allerdings nicht zu unterschätzenden organisatorischen Aufwand hinsichtlich der zeitlichen und räumlichen Ressourcen und der curricularen Einbindung. Dabei muss der Verzahnung monoprofessioneller und interprofessioneller Lehrinhalte besondere Aufmerksamkeit gewidmet werden.

## Introduction

Interprofessional Education (IPE) occurs when two or more professions learn from, with and about each other to improve collaboration and the quality of care [http://caipe.org.uk/resources/defining-ipe/], [1]. In direct connection with demographic shifts and the rise in complex diseases, increased cooperation between the skilled health professions and medicine is being repeatedly called for [1], [2], [3], [4]. Interprofessional teamwork should begin during professional training or university study [3]. Implementing IPE programs at educational institutions, universities of applied sciences and universities is at present very rare, but is receiving ever greater priority [3]. For instance, the National Competency-based Catalogue of Learning Objectives for Undergraduate Medical Study (NKLM) defines the ability to work in teams in multiprofessional settings as relevant educational content for those studying medicine [http://www.nklm.de].

The program, funded by the Robert Bosch Stiftung “Operation Team – Interprofessional Learning in the Health Care Professions,” focuses precisely on this to support eight cooperative projects across Germany between university medical schools, universities of applied sciences, and educational institutions providing vocational training in the skilled health professions (http://www.bosch-stiftung.de/content/language1/html/44080.asp). Among these projects is the “Interprofessional Practice in the Health Professions Project” (IPHiGen) between the Medical School of the Ruhr University Bochum (RUB) and the Department for Applied Health Sciences at the Hochschule für Gesundheit (hsg). This paper presents the knowledge and experience gained from organizing this project. The evaluation results will be published at a later date.

## Project description

“Interprofessional Practice in Health Care" (Interprofessionelles Handeln im Gesundheitswesen, IPHiGen) promotes interprofessional education for the students in the model degree program in medicine at RUB and the bachelor degree programs in nursing, physiotherapy, occupational therapy, speech therapy and midwifery at the hsg. In the hsg programs, students earn both a professional qualification by passing the state examination in a particular field and the academic degree of Bachelor of Science. The RUB model study program in medicine and the other five academic programs all focus on problem-solving, practice and needs orientation.

At the end of 2013 the cooperative project between RUB und hsg received a two-year grant from the Robert Bosch Stiftung. Following a preparatory phase, the first curricular units were offered in the 2014/15 winter semester and successfully completed in the 2015 summer semester. The course offerings encompassed a total of 32 hours divided over four learning sequences. A project group comprised of members from all of the professional groups involved assumed responsibility for development.

The aim of IPHiGen was to design and evaluate interprofessional teaching formats in order to create and implement joint educational structures between the bachelor programs at the hsg and the medical degree program at RUB.

The core competencies to be acquired were generated as part of an analysis carried out by the project group. Firstly, practice areas in the clinical setting (emergency care, acute inpatient care, rehab, etc.) were identified. However, these were not particularly suitable for designing an interprofessional teaching strategy. Therefore, the project group used learning fields and core competencies that matched with the international IPE requirements, such as the WHO Framework for Action on Interprofessional Education & Collaborative Practice [1] and the Core Competencies for Interprofessional Collaborative Practice [5]. The main focus of IPHiGen was placed on the acquisition of communication skills, knowledge about and appreciation of the work done by other health professions, teamwork, and reflection on one’s own professional role and responsibilities. Students worked primarily in small groups to enable direct exchange and sharing between the professional groups. The interprofessional student teams in the IPHiGen project were formed for a period of two semesters to build a culture of effective and trustworthy communication. Crucial to all of the learning sequences was the final reflection on the overall experience and what was learned. Invited to reflect by responding to specific questions, students were to identify the knowledge and experiences they had gained to strengthen their focus on the acquired competencies and the advantages of interprofessional collaboration. According the WHO [1] and Hammick et al. [6], experiences with interprofessional learning are particularly effective when the principles of adult learning, such as problem-based learning, are applied, when students interact with each other, and when the learning methods reflect practical experience. These educational principles were taken into account when designing the learning sequences.

After an introductory lecture on interprofessional practice, the students discussed interprofessional health care in small groups during the **first learning sequence**. They talked about their profession’s ideals, identified their reasons for studying, and presented each other the structure and content of their six study programs. In the second step, the students elaborated stereotypes and prejudices with the goal of critically questioning them by increased awareness. Interprofessional learning can positively influence these types of perceptions [7], [8], [9]. In doing this, each profession’s sphere of responsibility and activities were introduced and discussed, since a clear understanding of typical professional roles and responsibilities belongs to the core competencies of interprofessional practice [1], [10].

The **second learning sequence** covered the topic of patient safety. Based on personal experience from everyday life in health care, the students specified necessary factors for and potential barriers to successful interprofessional collaboration. By doing this, a direct attempt was made to reflect on experiences of routine work in an interprofessional setting [1].

In addition, the students acquired knowledge regarding the legal basis for their own professions. During a self-study period, the students read up on the professional codes and statutory regulations governing the practice of their professions and presented their findings in small groups. In doing this, they identified critical points of intersection that occur in interprofessional collaboration, discussed the decision-making strategies and workflow typical to each profession, and covered for each individual occupation the areas of responsibility and the professional limits that could endanger patient safety.

The **third learning sequence** focused on user orientation to spotlight well-coordinated and effective care in the inpatient, rehabilitative, and outpatient contexts. Using two case examples with real patients from neurology and social pediatrics, students first developed a uniprofessional plan for therapy and care. This plan then served as a basis for discussion in interprofessional small groups. The goal was to generate a joint interprofessional treatment and care plan. In the course of accomplishing this, the professional spheres of activity and responsibility discussed in the previous learning sequences were revisited as focal topics.

The **fourth learning sequence** focused on teamwork. In a joint plenum session for all students, experts from academia and practice discussed the patient cases from the third learning sequence. Afterwards, the students reflected on the experts’ approach within the context of the treatment and care plans they themselves had generated. In doing so, they focused particularly on reaching decisions as teams and pursuing a shared strategy for ensuring interprofessional patient care. As a result, the connection between theory and practice is established [1]. As a creative reflection on the project and based on their own experiences, the students proposed their own ideal teaching strategies for future IPE course offerings for RUB and hsg students. These proposals were presented in the form of a poetry slam and given awards as part of the final “IPE Slam”.

Over the entire course of the project, the interprofessional small groups were led by an instructor whose task it was to guide the students’ learning process and reflections on what they had learned. The instructors did not assume the traditional role of teacher or lecturer, but rather that of supportive mentor of the communication process. These instructors were trained in seminars regarding targeted preparation and follow-up work. Ultimately, the interprofessional education differed very significantly from the seminars in each of the individual professions [11]. In addition, these instructors took part in the reflection seminars to contemplate their own professional roles, to discuss how to handle difficulties between the different student groups, and to share their experiences with interprofessional education [6], [12].

Project evaluation was done using process data and learning results from the small groups, which were used to adapt the learning units as they took place. In addition, quantitative data was collected using an evaluation survey and the Readiness for Interprofessional Learning scale (RIPLS) [13] as a means to measure learning outcomes. The quantitative data will be published at a later date.

## Results

The project results should be presented and discussed within the context of logistical issues and questions concerning teaching methods.

**Figure 1:**
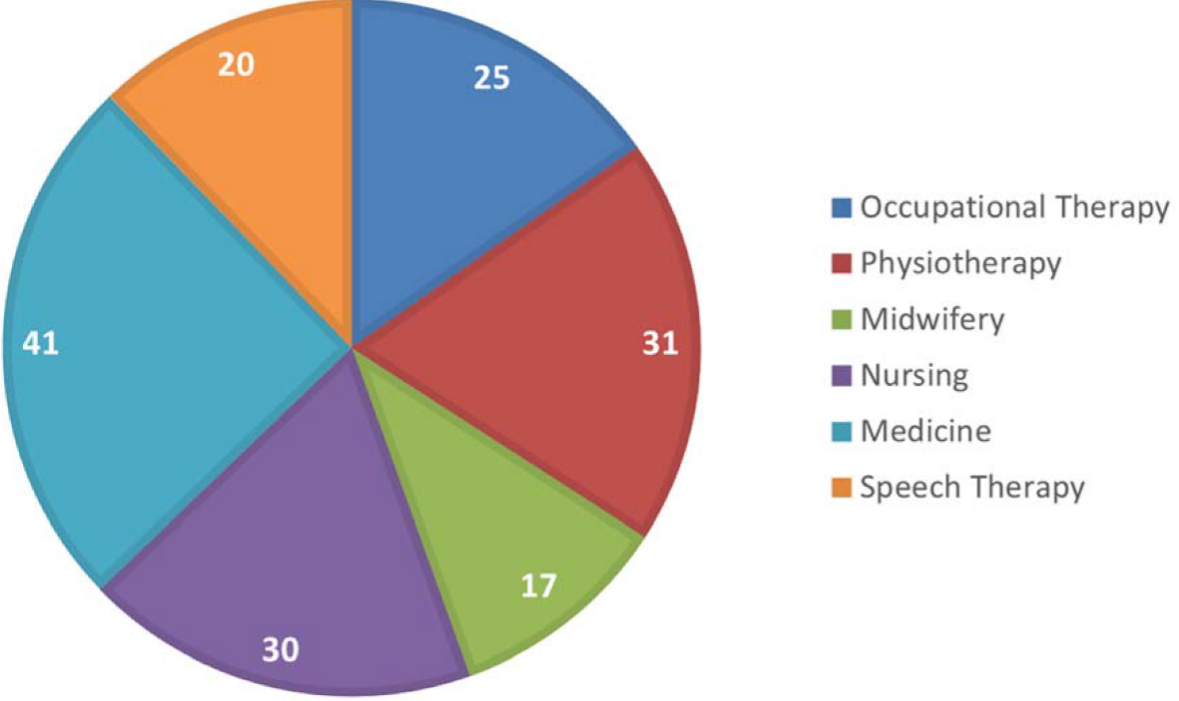
Number of students by course of studies

Overall, 220 students were involved in the project comprising medicine (n=41), nursing (n=38), physiotherapy (n=42), occupational therapy (n=34), speech therapy (n=31), and midwifery (n=34) (see figure 1 (Fig. 1)). The medical students were in their ninth semester of study at the start of the project, the hsg students in their third. Although the students had different degrees of theoretical knowledge at this point, both the hsg students and the medical students had gained similar practical experience as the hsg students have a high degree of practical training from the very start of their programs.

The lessons during the project were integrated into the curriculum of each study program as a mandatory course, although the attendance requirements were handled differently. The medical students were required to obtain written confirmation of attendance from the instructors; otherwise repeats or substitute work would have been necessary. Students at the hsg were bound to the attendance rules laid down for each profession.

**Figure 2:**
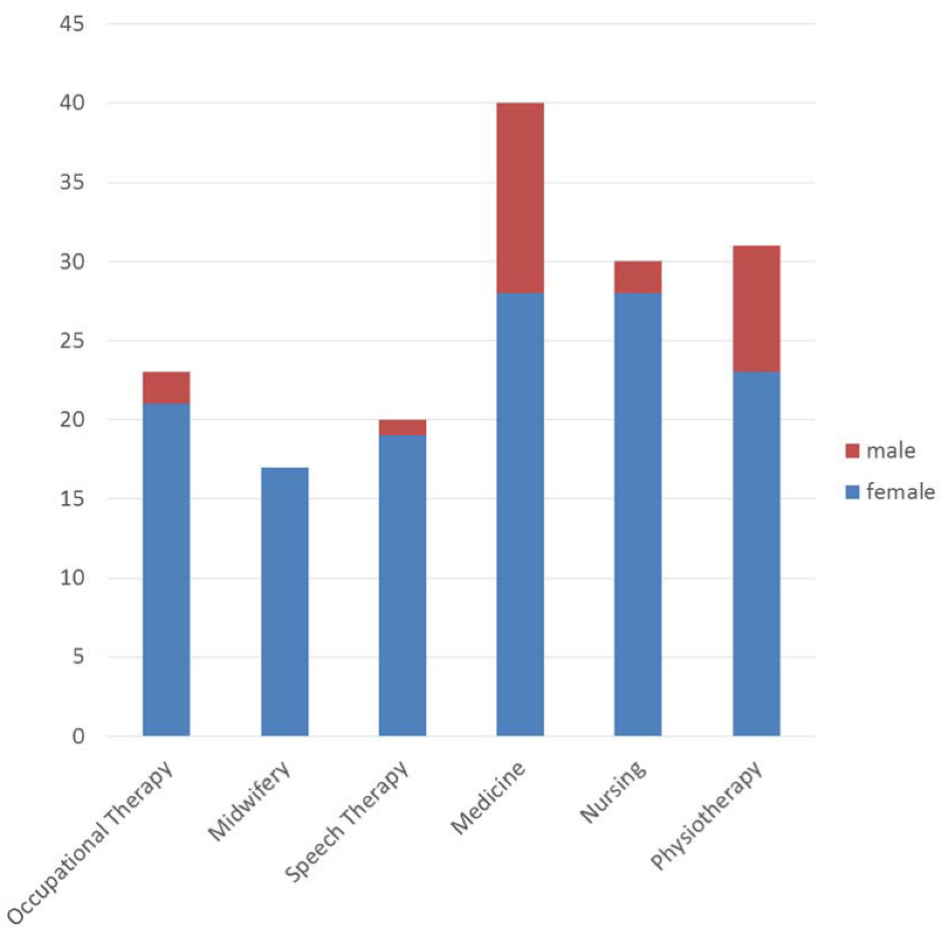
Course of studies by sex

At the beginning of the project, a total of 164 students filled in a questionnaire about their professional experience and experience with interprofessional collaboration. This included 25 male and 135 female students; four students did not answer this question. The high percentage of female students was mainly due to the distribution in hsg the study programs. Only 12% of the hsg participants are male; among the medical students the percentage is 30% (see figure 2 (Fig. 2)). In the group of medical students there is also a higher percentage of over-30 year-olds (31.7% vs. 4.8% of hsg students). In addition, the physicians-to-be possess more prior professional experience, for instance as a result of completed professional educational programs (43.9% vs. 21.1%). Almost all of the students (93.9%) reported that they had already gathered experience in the area of interprofessional collaboration, 43.3% through participation in projects, 66.5% through attending courses, 27.4% through previous vocational training, and 74.4% through internships. Multiple responses were possible.

In the final project evaluation, 86.5% of the 104 respondents were of the opinion that it is important that all students in the six professional groups participate in the project. Single professions were sometimes missing from the small groups. Reasons for non-participation were incompatible schedules due to overlapping practical study phases (n=15) and holidays (n=19), lack of a requirement to participate (n=5), and lack of motivation (n=5). For 32.7% of the students the need to travel between campuses in order to attend the sessions was a problem. Absences were critically noted by the students in their open-ended responses (n=27) since an interprofessional project only makes sense if all of the professions playing a role in health care are present.

Specifically the chance to share knowledge and become acquainted with each other, to work together on patient cases, and to discuss the experiences with interprofessional collaboration in practice were positively evaluated. In terms of future projects, students expressed a desire for an even closer focus on practice, for instance collaboration on a patient case for which the process of providing care is not yet complete and still requires much decision-making and action.

## Discussion

As part of the cooperative IPHiGen project, students in occupational therapy, midwifery, speech therapy, medicine, nursing and physiotherapy had the opportunity to exchange information about their professional roles, responsibilities and regulations, to reflect on stereotypes and prejudices, and to develop joint treatment strategies for a patient case, as well as to discuss possible solutions to problems encountered in the provision of health care.

## Structural aspects

With the participation of six study programs there were challenges especially in coordinating schedules. Program-specific phases for theoretical or practical education must be taken into account in the overall planning and scheduling. Five windows of time existed in the regular course schedules for the six study programs in which the joint courses could be synchronized, three of these in the winter semester and two in the summer semester. These windows offered sufficient possibility for the students to share and become familiar with each other. There was sufficient time to hold group discussions and work on joint tasks. Along with coordinating scheduled meeting times, it was also the challenge of finding adequate spaces in which to hold the interprofessional seminars during the class period. This required a great degree of coordination and cooperation between the universities. Lack of space was the reason why not all learning sequences could take place at one location.

Due to overlapping schedules and differing attendance rules, there was a difference in participant numbers in the individual seminar groups. In the literature it is clear [6] that administrative aspects in particular influence participation in interprofessional courses. Specifically the varying rules regarding attendance and schedule overlaps with other academic phases and university holidays led to the fact that during the IPHiGen project not all of the professions were represented at all times, something that compromised the interprofessional interactions and work. Kilminster et al. [13] and Carpenter [7] assert that different rules about course attendance need not be a problem. However, Carpenter [7] refers to a one-day workshop and not, as in this case, to seminars that are scheduled to take place over two semesters. In a qualitative study by Altin et al. [14] on conducting interprofessional training, it became clear that different attitudes among participants is a great problem when implementing interprofessional courses.

The students differed greatly in terms of sociodemographic profiles and prior knowledge. This had an influence on the discussions, since, due to their advanced study, the medical students had better theoretical knowledge and more practical expertise as a result of also having had more previous professional experience, for instance from nursing. For future projects, the educational level of the different student groups should be analyzed more carefully in regard to setting educational goals, formulating course assignments, and the learning objectives.

In respect to future implementation, the conclusion can be drawn that the logistical aspects must be considered very early on and very thoroughly during the planning phase.

## Methodological challenges

The learning sequences were designed by an interprofessional project group. As a consequence, communication with and between the various study programs was meant to be ensured, and each of the professional groups was involved in the planning and decision-making processes.

Different educational formats were used. Interactive learning formats were tried out that allow for a reflective discussion culture between the professional groups. Some studies have already been able to document the success of interprofessional small groups ([15], [16], [17]). Trained instructors who guided the interprofessional groups ensured that students reflected on what they had learned. Lachmann et al. [18] were able to show that continual response to questions encouraging reflection contributes to a better understanding of interprofessional teamwork. Domac et al. [19] view reflection as the single decisive factor for learning success. The IPHiGen reflective questions also played a role in process control and significantly affected further development of the project.

An important aspect in designing the learning sequences was the synthesis of subject-specific, monoprofessional content with the development of interprofessional competencies. This should be seen particularly in the group work on the case example, in which the students should, in alternating mono- and interprofessional learning phases, discuss not only the case, but also the results from their own occupational perspectives in the small interprofessional groups. The language specific to each profession was also identified during the learning sequences as a possible inhibitor of interprofessional collaboration (see [20], [10]). Some of the professional groups refer to the affected person as “patient”, others as “client”, and even others as a “person in need of care” or none of these designations. In this case, acceptable terms must be found by all involved. Different tasks and responsibilities necessitate different approaches to professional work. This problem was solved through discussion of two very different, real cases.

## Conclusion

The challenges facing interprofessional education exist on two levels. On the one hand, IPE requires an adequate organizational framework, such as rules concerning attendance, along with the coordination of academic schedules and classroom space. On the other hand, monoprofessional and interprofessional subject matter and teaching methods should be coordinated with each other so that interprofessionalism becomes a self-evident component of professional education and training.

## Funding

The project is received a grant from the Robert Bosch Stiftung (project number 32.5.1316.0009.0)

## Acknowledgements

We wish to thank all the patients, experts, instructors and students for their active participation.

## Competing interests

The authors declare that they have no competing interests.

## References

[1] World Health Organization (2010). Framework for Action on Interprofessional Education & Collaborative Practice.

[2] Wissenschaftsrat (2012). Empfehlungen zu hochschulischen Qualifikationen für das Gesundheitswesen.

[3] Walkenhorst U, Mahler C, Aistleithner R, Hahn E, Kaap-Fröhlich S, Karstens S, Reiber K, Stock-Schröer B, SDottas B (2015). Positionspapier GMA-Ausschuss - "Interprofessionelle Ausbildung in den Gesundheitsberufen". GMS Z Med Ausbild.

[4] Robert-Bosch-Stiftung (2011). Ausbildung für die Gesundheitsversorgung von morgen.

[5] Interprofessional Education Collaborative Expert Panel (2011). Core competencies for interprofessional collaborative practice: Report of an expert panel.

[6] Hammick M, Freeth D, Koppel I, Reeves S, Barr H (2007). A best evidence systematic review of interprofessional education: BEME Guide no. 9. Med Teach.

[7] Carpenter J (1995). Interprofessional education for medical and nursing students: evaluation of a programme. Med Educ.

[8] Hind M, Norman I, Cooper S, Gill E, Hilton R, Judd P, Jones SC (2003). Interprofessional perceptions of health care students. J Interprof Care.

[9] Lewitt MS, Ehrenborg E, Scheja M, Brauner A (2010). Stereotyping at the undergraduate level revealed during interprofessional learning between future doctors and biomedical scientists. J Interprof Care.

[10] Suter E, Arndt J, Arthur N, Parbossingh J, Taylor E, Deutschlander S (2009). Role understanding and effective communication as core competencies for collaborative practice. J Interprof Care.

[11] Reeves S, Freeth D (2002). The London training ward: an innovative interprofessional learning initiative. J Interprof Care.

[12] Mahler C, Rochon J, Karstens S, Szecsenyi J, Hermann K (2014). Internal consistency of the readiness for interprofessional learning scale in German health care students and professionals. BMC Med Educ.

[13] Kilminster S, Hale C, Lascelles M, Morris P, Roberts T, Stark P, Sowter J, Thistlethwaite J (2004). Learning for real life: patient-focused interprofessional workshops offer added value. Med Educ.

[14] Altin SV, Tebest R, Kautz-Freimuth S, Redaelli M, Stock S (2014). Barriers in the implementation of interprofessional continuing education programs--a qualitative study from Germany. BMC Med Educ.

[15] Ruebling I, Pole D, Breitbach AP, Frager A, Kettenbach G, Westhus N, Kienstra K, Carlson J (2014). A comparison of student attitudes and perceptions before and after an introductory interprofessional education experience. J Interprof Care.

[16] Darlow B, Coleman K, McKinlay E, Donovan S, Beckingsale L, Gray B, Neser H, Perry M, Stanley J, Pullon S (2015). The positive impact of interprofessional education: a controlled trial to evaluate a programme for health professional students. BMC Med Educ.

[17] Meffe F, Moravac C, Espin S (2012). An interprofessional education pilot program in maternity care: findings from an exploratory case study of undergraduate students. J Interprof Care.

[18] Lachmann H, Fossum B, Johansson UB, Karlgren K, Ponzer S (2014). Promoting reflection by using contextual activity sampling: a study on students' interprofessional learning. J Interprof Care.

[19] Domac S, Anderson L, O'Reilly M, Smith R (2015). Assessing interprofessional competence using a prospective reflective portfolio. J Interprof Care.

[20] Hall P (2005). Interprofessional teamwork: professional cultures as barriers. J Interprof Care.

